# Hydrogen-Tolerant La_0.6_Ca_0.4_Co_0.2_Fe_0.8_O_3–*d*_ Oxygen Transport Membranes from Ultrasonic Spray Synthesis for Plasma-Assisted CO_2_ Conversion

**DOI:** 10.3390/membranes13110875

**Published:** 2023-11-07

**Authors:** Aasir Rashid, Hyunjung Lim, Daniel Plaz, Giamper Escobar Cano, Marc Bresser, Katharina-Sophia Wiegers, Giorgia Confalonieri, Sungho Baek, Guoxing Chen, Armin Feldhoff, Andreas Schulz, Anke Weidenkaff, Marc Widenmeyer

**Affiliations:** 1 Research Division of Materials & Resources, Technical University of Darmstadt, Peter-Grünberg-Str. 2, 64287 Darmstadt, Germany; hyunjung.lim@stud.tu-darmstadt.de (H.L.); sungho.baek@stud.tu-darmstadt.de (S.B.); anke.weidenkaff@mr.tu-darmstadt.de (A.W.); 2 Institute for Materials Science, University of Stuttgart, Heisenbergstr. 3, 70569 Stuttgart, Germany; 3 Institute of Physical Chemistry and Electrochemistry, Leibniz University Hannover, Callinstr. 3A, 30167 Hannover, Germany; giamper.escobar@pci.uni-hannover.de (G.E.C.); armin.feldhoff@pci.uni-hannover.de (A.F.); 4 Institute of Interfacial Process Engineering and Plasma Technology (IGVP), University of Stuttgart, Pfaffenwaldring 31, 70569 Stuttgart, Germany; marc.bresser@igvp.uni-stuttgart.de (M.B.); katharina.wiegers@igvp.uni-stuttgart.de (K.-S.W.); andreas.schulz@igvp.uni-stuttgart.de (A.S.); 5 ESRF—European Synchrotron Research Facility, 71 Avenue des Martyrs, 38043 Grenoble, France; 6 Fraunhofer Research Institution for Material Recycling and Resource Strategies IWKS, Brentanostr. 2A, 63755 Alzenau, Germany; guoxing.chen@iwks.fraunhofer.de

**Keywords:** oxygen transport membranes, CO_2_ conversion, H_2_ tolerance, ultrasonic spray synthesis, plasma-assisted process

## Abstract

La_0.6_Ca_0.4_Co_1–*x*_Fe*_x_*O_3–*d*_ in its various compositions has proven to be an excellent CO_2_-resistant oxygen transport membrane that can be used in plasma-assisted CO_2_ conversion. With the goal of incorporating green hydrogen into the CO_2_ conversion process, this work takes a step further by investigating the compatibility of La_0.6_Ca_0.4_Co_1–*x*_Fe*_x_*O_3–*d*_ membranes with hydrogen fed into the plasma. This will enable plasma-assisted conversion of the carbon monoxide produced in the CO_2_ reduction process into green fuels, like methanol. This requires the La_0.6_Ca_0.4_Co_1–*x*_Fe*_x_*O_3–*d*_ membranes to be tolerant towards reducing conditions of hydrogen_._ The hydrogen tolerance of La_0.6_Ca_0.4_Co_1–*x*_Fe*_x_*O_3–*d*_ (*x* = 0.8) was studied in detail. A faster and resource-efficient route based on ultrasonic spray synthesis was developed to synthesise the La_0.6_Ca_0.4_Co_0.2_Fe_0.8_O_3–*d*_ membranes. The La_0.6_Ca_0.4_Co_0.2_Fe_0.8_O_3–*d*_ membrane developed using ultrasonic spray synthesis showed similar performance in terms of its oxygen permeation when compared with the ones synthesised with conventional techniques, such as co-precipitation, sol–gel, etc., despite using 30% less cobalt.

## 1. Introduction

Materials production is one of the major sources of greenhouse gas emissions; it accounts for around 25% of anthropogenic CO_2_ emissions [1,2]. There is indeed a need for techniques to store and utilise CO_2_ emissions, and different approaches have already been investigated [3,4,5]. An emerging CO_2_ conversion technology that needs more attention and that, to a large extent, considers sustainability criteria as well is plasma-assisted CO_2_ conversion [6,7,8]. This technique can efficiently produce CO and O_2_ from fossil CO_2_ emissions that would otherwise emit into the environment using oxygen transport membranes [6]. Oxygen transport membranes gained a good amount of attention in the recent years due to their potential of assisting with CO_2_ reduction [9,10,11,12,13,14]. Mixed ionic electronic conducting (MIEC) materials are prominent for their transport properties [15,16,17,18]. As oxygen transport membranes, these materials have shown significant performance in terms of oxygen permeation [6,19]. Perovskite-type oxide membranes are emerging as one of the favoured options for enhancing the CO_2_ conversion due to their excellent oxygen permeation, but they are often lacking in terms of CO_2_ tolerance [20,21,22]. La_0.6_Ca_0.4_Co_1–*x*_Fe*_x_*O_3–*d*_ (LCCF) membranes, however, have been shown to be CO_2_-tolerant, and they provide excellent oxygen permeation, as proven in our previous works [6,19]. With the goal of incorporating green hydrogen (H_2_) into the CO_2_ conversion process, this work takes a step further by investigating the compatibility of LCCF membranes with H_2_ fed into the plasma. This will enable plasma-assisted conversion of the CO produced in the CO_2_ reduction process into green fuels, like methanol. This can be made possible by reacting the CO, obtained from CO_2_ splitting in the plasma, with green H_2_. To achieve this conversion, the oxygen concentration needs to be kept in check in the plasma system to avoid recombination to CO_2_. This can be achieved with the CO_2_-tolerant oxygen transport membranes, and it has been successful so far based on our previous works [6,19]. However, the introduction of H_2_ demands that the CO_2_-tolerant membranes are H_2_-tolerant and that they exhibit similar performance in terms of oxygen permeation. The selection of oxygen membrane materials for CO_2_ conversion requires high oxygen permeability that is adequate enough to be suitable for industrial use, chemical stability, thermal stability in CO_2_ and H_2_ atmospheres, and suitable mechanical properties [23,24,25,26]. The material system that is of constant interest to our work is the LCCF single-phase perovskite-type oxides [19]. This material system in its different Co:Fe ratios has been shown to be thermally stable against CO_2_, and it also provides an oxygen permeability (1.3 mL∙min^−1^∙cm^−2^∙mm) for Co:Fe that equals 1:1, which is of industrial standard when used as a hollow fibre [6]). This work focusses on one of the LCCF variants, La_0.6_Ca_0.4_Co_0.2_Fe_0.8_O_3–*d*_ (LCCF_6428), to investigate its behaviour against H_2_, thereby allowing lesser usage of Co without sacrificing the oxygen permeation performance.

LCCF possesses a combination of a rare earth element (La) and another critical element (Co), implying that a high value waste is expected once the membrane reaches its end of life. A critical raw material as per the EU classifications [27] implies a high supply risk is associated with the material and it is economically important. The Herfindahl–Hirschman Indices (HHI) for La and Co are greater than 2500, indicating a highly concentrated marketplace for the availability of these elements [28,29]. One of the known ways to address criticality is an emphasis on substitution, which has been used to reduce the usage of critical elements without sacrificing much of the performance, as observed in the further sections. A better and lasting approach to tackle criticality is the recycling of materials, and this is indeed of interest to our work. The recycling of the perovskite-type oxide membranes is in progress, and it will be covered in detail in a future publication.

A challenging task, however, for current materials-science-based research is to figure out pathways that can allow for a sustainable membrane material synthesis while also ensuring resource efficiencies and environmental friendliness. This can be addressed by making use of ultrasonic spray synthesis (USS). USS is preferred over conventional techniques, such as co-precipitation (CP) or sol–gel, due to it holding numerous advantages. USS is a one-pot, solution-based synthesis technique used to synthesise inorganic nanomaterials. Its mechanism is based on the nebulisation of the liquid precursor initiated by the ultrasonic waves, thus driving the generated precursor aerosol into the heated zone and collecting the powder product in the extraction system. It enables morphological and compositional control of the product. Due to shorter diffusion lengths imposed by the size of the enclosed droplets, this technique enables faster and homogeneous growth of particles [30]. It is considerably faster, and it has a greater scope of being a continuous process. It is a simple synthesis method requiring just deionised (DI) water as a solvent in most cases, which helps in keeping the direct process emissions low, unlike sol–gel or CP, where additional complexing agents may be required, such as citric acid, ethylenediaminetetraacetic acid, etc. [19,31], thereby leading to higher direct process emissions. Looking specifically at the CP synthesis of LCCF, additional ammonium carbonate is needed for the precipitation reaction [19]. USS often does not require an additional calcination step, as the high-temperature pyrolysis pretty much accounts for it. This coupled with lower process emissions and its scope of being a continuous process can prove very crucial in terms of increasing the resource efficiency of the synthesis.

This work is focused on the synthesis of LCCF_6428 membranes using USS and establishing their tolerance against H_2_. The behaviour of the membrane in a CO_2_/H_2_ plasma was also studied.

## 2. Materials and Methods

### 2.1. Membrane Synthesis

The synthesis of LCCF_6428 membrane materials was carried out with USS. A detailed description of the USS setup can be accessed in the Supplementary Material Section S1.

The respective metal nitrates were used as received (namely, La(NO_3_)_3_·6H_2_O (99.9% thermo scientific, Kandel, Germany), Ca(NO_3_)_2_·4H_2_O (99.0–103.0%, thermo scientific, Kandel, Germany), Co(NO_3_)_2_·6H_2_O (98.0–102.0%, thermo scientific, Kandel, Germany), and Fe(NO_3_)_3_·9H_2_O (98.0–101.0%, Alfa Aesar, Kandel, Germany).

**Figure 1:**
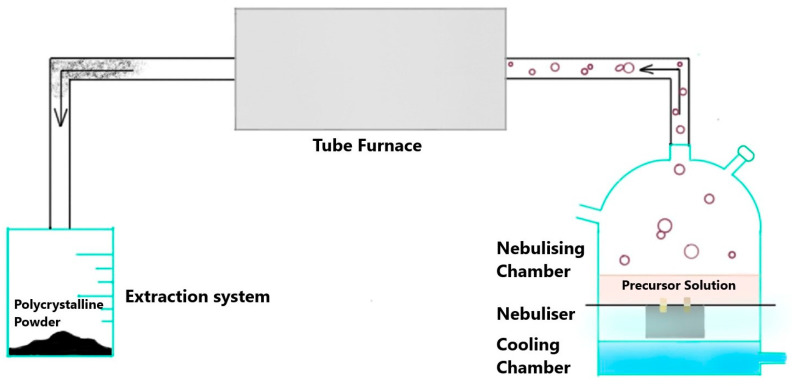
Schematics of ultrasonic spray synthesis (USS).

Weighed amounts of the metal nitrates were dissolved in DI water to prepare a 1M precursor solution (0.05 moles in 50 mL). The precursor solution was then added to the nebulising chamber of the home-made USS setup (Figure 1) with a thin transparent foil separating the solution from the nebuliser, which generates ultrasonic waves that convert the precursor solution into tiny droplets. An inert gas (argon) supply with a flowrate of 2000 sccm was also attached to the setup. The droplets are transported into the heated tube furnace (900 °C) by the inert gas in the form of an aerosol. Each aerosol droplet undergoes repeated precipitation reactions individually followed by pyrolysis, leading to the formation of a product particle. These particles coalesce together, leading to the formation of polycrystalline solid powder particles [31]. The powder product was obtained with a maximum production rate of 2.5 g/h. The schematics of the process can be visualised in Figure 1.

The obtained powder was dried in an oven at 100 °C for 30 min. The dried powder was afterwards uniaxially pressed in a stainless-steel mould (16 mm diameter) with a force of ~20–25 kN, thus producing disc-shaped membranes (polished to ~1 mm in thickness). The membranes were then sintered at 1180 °C for 30 h in a muffle furnace with a heating and cooling rate of 3 K∙min^−1^. From the Archimedes density measurements, the membranes exhibit a relative density of approximately 89%.

### 2.2. Material Characterisation

The structural analysis of the powdered samples after sintering was carried out with high-flux high-resolution powder X-ray diffraction using a high-resolution powder diffraction beamline (*λ* = 0.427 Å) at ESRF (ID22) [32]. A two-dimensional Dectris Eiger2 X 2M-W CdTe pixel detector was mounted on the arm of the diffractometer. At each nominal 2*θ* value, a two-dimensional image was recorded showing 512 distinct regions of interest (defined size 1 × 20 pixels, h × v) for each of the 13 analyser crystals. FullProf.2k (version: 7.70) was used to carry out the Rietveld refinements. The reflection profile was fitted by pseudo-Voigt functions. Additionally, a step-by-step crystal structure investigation of samples at each stage of the synthesis was carried out using a STOE STADI MP X-ray diffractometer (STOE, Darmstadt, Germany) with Mo-*Kα*_1_ radiation. Initially, powder X-ray diffraction in transmission mode was employed straight after obtaining the powder, and then another measurement using reflection mode was recorded after the powders were pressed into disc-shaped membranes and sintered. The measurement was repeated after the membranes were subjected to an oxygen permeation test and a plasma test. Morphological studies of the polished, sintered, fresh membrane were performed using VEGA3-TESCAN (TESCAN GmbH, Dortmund, Germany) scanning electron microscope (SEM) using a beam voltage of 20 kV and to assess the uniformity of the material composition. Subsequent energy dispersive X-ray spectroscopy (EDXS) mapping analysis, with a beam voltage of 30 kV, was carried out at five different areas of the membranes. The non-polished plasma-exposed membrane was analysed in a similar manner as described before using a Philips XL30 FEG (Koninklijke Philips N.V, Eindhoven, The Netherlands) and EDAX Genesis (AMATEK GmbH, Unterschleissheim, Germany) EDXS detector. Moreover, inductively coupled plasma optical emission spectroscopy (ICP-OES), using a PerkinElmer Optima 8300 (PerkinElmer, Rodgau, Germany), was also employed to verify the material composition. The sample solution for ICP-OES was prepared by dissolving the sample in diluted nitric acid. The measurement was performed two times. For measuring the oxygen content, hot gas extraction analysis (HGE), using carbothermal fusion of the material in helium gas atmosphere on a LECO ONH836 (LECO Instrumente GmbH, Mönchengladbach, Germany), was carried out. The measurement was performed three times. The stability in reducing conditions of H_2_ was studied with Thermo-Gravimetric Analysis (TGA) and Differential Scanning Calorimetry (DSC). TGA was carried out using a NETZSCH STA 449F3 (NETZSCH-Gerätebau GmbH, Selb/Bayern, Germany) thermal analyser. The measurement consists of heating 40–60 mg of sample powder in an alumina crucible at a heating rate of 10 K∙min^−1^ up to 1100 °C in a 95-vol.% Ar-5-vol.% H_2_ (purity 99.999%) atmosphere maintaining a gas flow rate of 60 mL∙min^−1^. Argon (purity 99.999%) was supplied in parallel through a separate valve at 20 mL∙min^−1^ throughout the measurement. DSC measurements were carried out using a NETZSCH DSC 404 C (NETZSCH-Gerätebau GmbH, Selb/Bayern, Germany). The measurement consists of heating 20–30 mg of sample powder on an alumina pan at 800 °C with a heating rate of 10 K∙min^−1^ in 95-vol.% Ar-5-vol.% H_2_ (purity 99.999%). Additional details related to the composition and purity of gases can be accessed in the Supplementary Materials Section S4. The preliminary plasma studies were carried out using a self-made plasma reactor at a temperature of 1130 °C (heating rate > 1000 K∙s^−1^) with an inlet flow consisting of 6.7 slm CO_2_ (purity 99.995%) and 1 slm H_2_ (purity 99.999%) for 1 h. The relative humidity inside the plasma reactor was close to 100%.

### 2.3. Oxygen Permeation Measurements

Oxygen permeability measurements were conducted using a home-made high-temperature permeation setup, which has been described in detail elsewhere [33]. The LCCF membranes were sealed on an alumina tube with a commercial ceramic sealant (Huitian 276, Shenyang, China). Synthetic air (20 vol.% O_2_ and 80 vol.% N_2_) was used as feed gas at a rate of 150 mL∙min^−1^, and CO_2_ was used as the sweep gas at a rate of 29 mL∙min^−1^. The measurements were performed at 900 °C for 12 h. An online-coupled Agilent 7890A gas chromatograph (Agilent Technologies, Shanghai, China) equipped with a Carboxen^®^ 1000 (Sigma Aldrich Chemie GmbH, Taufkirchen, Germany) column was employed to analyse the gas mixture. The absolute flow rate was determined using neon with a flow rate of 1 mL∙min^−1^ as an internal standard. The total O_2_ leakage was calculated by measuring the N_2_ concentration and subtracted from the total O_2_ flux. Additional details related to the composition and purity of the gases can be accessed in the Supplementary Materials Section S4.

The potential leakages can be accounted for with the help of N_2_ leakage flux. This can be measured using the following Equation (1), assuming the leakage is in accordance with the Knudsen diffusion [34]:

(1) $$J_{N_{2}}^{Leak}:J_{O_{2}}^{Leak}=\sqrt{32/28\;}\;\times \;\frac{0.79}{0.21}=4.02$$

The oxygen permeation flux ($J_{O_{2}}$) can be determined using the following Equation (2):

(2) $$J_{O_{2}}\left(\frac{ml}{\min{cm}^{2}}\right)=\left(C_{O_{2}}-\frac{C_{N_{2}}}{4.02}\right)\times \frac{f}{S}$$

where $C_{O_{2}}\;$ is the measured concentration of oxygen, $C_{N_{2}}$ is the measured concentration of nitrogen on the sweep side, *F* is the flow rate and *S* is the effective membrane surface.

## 3. Results

### 3.1. XRD

**Figure 2:**
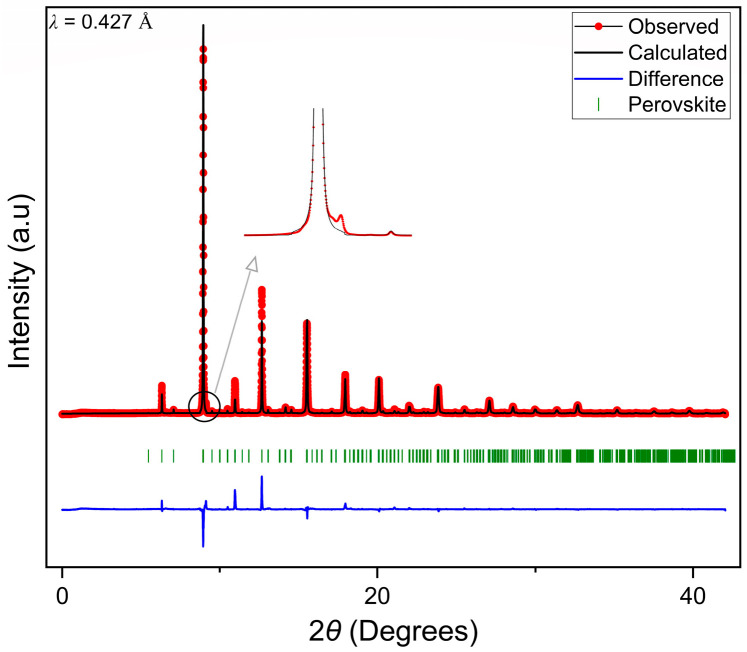
Refined XRD pattern of LCCF_6428 (*λ* = 0.427 Å) data collected in reference [35].

The XRD measurements were carried out using high-flux high-resolution powder X-ray diffraction. Figure 2 represents the XRD pattern for LCCF_6428 membrane prepared using USS followed by sintering at 1180 °C for 30 h. The pattern indicates proper crystallinity, and it can be associated with that of a single-phase perovskite structure. There is a presence of some minor impurity peaks as well, especially the one present at a lower angle of 2*θ* = 9°, as highlighted in Figure 2. This very low-intensity reflection is below the detection limit of the laboratory STOE STADI MP X-ray diffractometer (Mo-*Kα*_1_). Nevertheless, this was studied, and it can be linked to a Ruddlesden–Popper or a layered perovskite-type structure, though inconclusively. Regardless, we assume these impurities do not have any major impact on the structure or performance of the membrane. This will be discussed later in Section 3.4, Section 3.5, Section 3.6. The pattern was refined following a *Pnma* orthorhombic space group type. However, the Rietveld refinements indicate a deviation towards a lanthanum-rich composition from the expected composition with respect to the lanthanum and calcium content. Further, chemical analysis was performed (Section 3.3) to investigate its significance. More details about the XRD results and Rietveld refinements can be accessed in the Supplementary Material Section S2.

### 3.2. SEM

**Figure 3:**
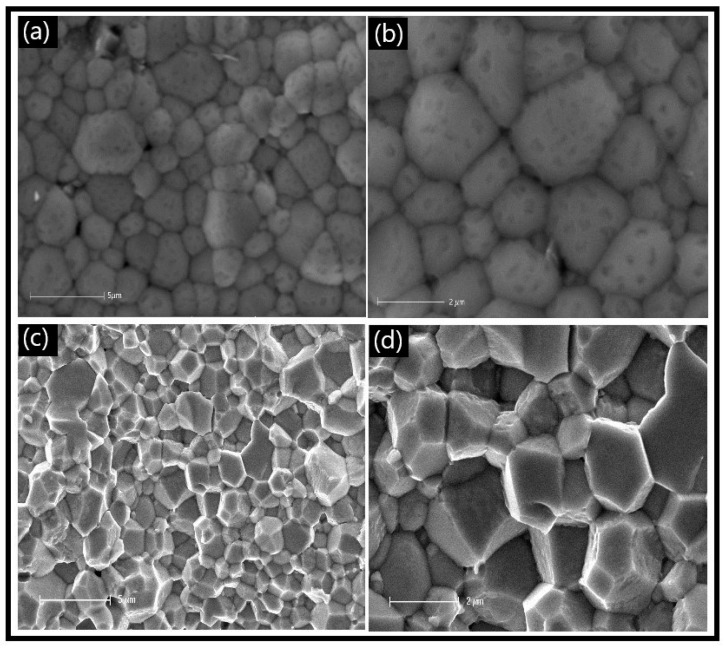
SEM micrographs of primary LCCF_6428 membrane. (**a**,**b**) Surface view and (**c**,**d**) cross-sectional view.

The SEM images of the LCCF_6428 membrane (Figure 3) revealed compactly arranged grains on the surface (Figure 3a,b), which is supported by the ~89% relative density measured, as mentioned earlier. A cross-sectional view (Figure 3c,d) also depicts the grains to be compact. There is a presence of some pores on the surface (Figure 3a,b), but they are isolated and do not appear to be coalescing together to cause any potential cracks.

### 3.3. Chemical Analysis

#### 3.3.1. EDXS

**Figure 4:**
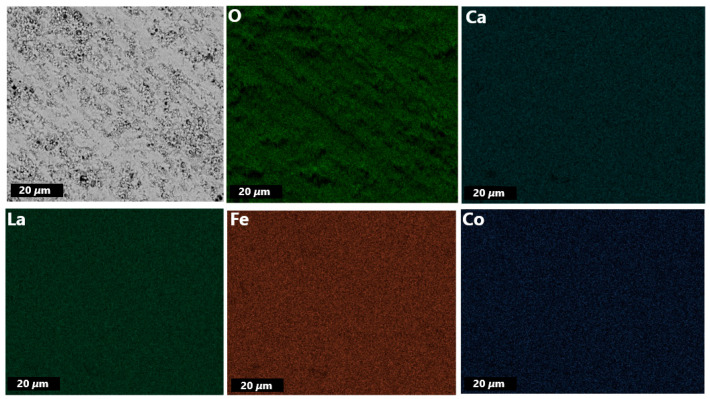
EDXS mapping of primary LCCF_6428 membrane (polished).

The EDXS mapping of the membrane disc (Figure 4) indicates homogeneous distribution of elements within the given spatial resolution across the membrane, which is further supported by EDXS elemental analysis (Section S3).

#### 3.3.2. ICP-OES

Regarding the observed difference between the refined composition and the expected composition, an ICP-OES analysis was performed. The results are summarised in Table 1. The ICP-OES results finally clarified that the elements are very close to the theoretically expected values, and they are even mostly within the measurement error bars.

#### 3.3.3. Hot Gas Extraction Analysis

The HGE analysis of LCCF_6428 (Table 2) revealed an oxygen content lower than three, which was used as the theoretical reference point.

The chemical analysis results indicate the chemical composition to be matching with the intended composition, i.e., La_0.6_Ca_0.4_Co_0.2_Fe_0.8_O_3–*d*_ with *d* = 0.12 ± 0.04.

### 3.4. Thermal Analysis

**Figure 5:**
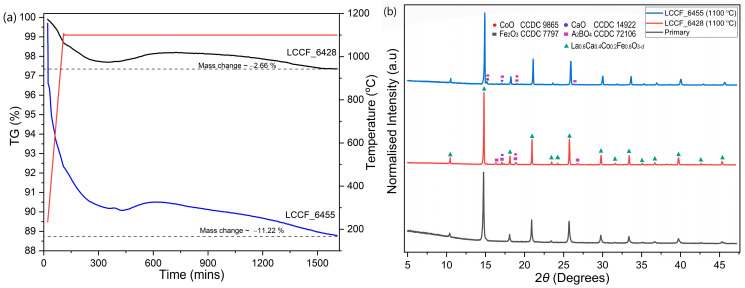
(**a**) TGA plots of LCCF_6455 and LCCF_6428 powder samples up to 1100 °C in 95 vol.% Ar-5 vol.% H_2_ for 25 h and (**b**) XRD plots of LCCF-6455 and LCCF_6428 powder samples after exposure to 95 vol.% Ar-5 vol.% H_2_ at 1100 °C vs. primary LCCF_6428 (Mo-*Kα*_1_).

In order to assess the thermal stability of LCCF membrane materials in CO_2_ and H_2_ atmospheres, thermogravimetric analysis (TGA) was carried out. The thermal stability of LCCF membranes against CO_2_ was already established in our previous work [19]. In this work, emphasis was laid on the H_2_ tolerance of LCCF. The thermogravimetric results, carried out in 95 vol. % Ar-5 vol. % H_2_ at 1100 °C isothermally over 25 h for La_0.6_Ca_0.4_Co_1–*x*_Fe*_x_*O_3–_*_d_* (*x* = 0.5) or LCCF_6455 and La_0.6_Ca_0.4_Co_1–*x*_Fe*_x_*O_3–_*_d_* (*x* = 0.8) or LCCF_6428, indicate a mass change of −11.22% and −2.66%, respectively (Figure 5a). The mass change for the LCCF_6455 sample was significantly higher than for LCCF_6428. This mass change can be associated to the Co content used and the subsequent oxygen loss in the material. It is known that in reducing conditions, the degree of reduction for a cobalt-doped perovskite-type oxide, such as La_0.6_Sr_0.4_Co_1−*x*_Fe*_x_*O_3–*d*_, increases with the increasing Co content [36]. The degree of impact of the mass change on the oxygen content of the perovskite-type oxide, which may affect its structure, can be estimated using relative mass change calculations. It is assumed that a perovskite-type oxide is able to retain a perovskite-type structure if the oxygen content is maintained within *d* = 0–0.5 [37]. However, the mass change calculations do not account for the possible phase transitions that can be induced in the material at higher temperatures when exposed to reducing conditions. It is, however, known that perovskite-type and structurally related oxides, such as (La,Sr)(Fe,Co)O_3_ and LaSrFeO_4_, undergo partial phase transitions after reduction in hydrogen and above 1000 K [38]. The XRD patterns of LCCF_6455 and LCCF_6428 (Figure 5b), after the H_2_ exposure at 1100 °C, might appear alike to some extent with the presence of secondary phases that are more pronounced in LCCF_6455. Given the considerable difference in mass change values, the similarities in the XRD patterns necessitated further investigation. Morphological studies revealed microstructural damage in LCCF_6455 (Figure S7). EDX analysis showed a significant cobalt deficiency in the H_2_-exposed LCCF_6455, with the elemental composition very close to LCCF_6428 (Table S3). This could indicate LCCF_6455 becoming similar to LCCF_6428 compositionally during the ongoing H_2_ exposure and, hence, it could be one of the reasons for the similarities noticed in the XRD patterns. As such, due to very high mass change (Figure 5a) and structural degradation, the higher Co content variant, i.e., LCCF_6455, was not favoured. The mass change calculations for LCCF_6428 can be better realised from the following Equation (3):

La_0.6_Ca_0.4_Co_0.2_Fe_0.8_O_2.88_ → La_0.6_Ca_0.4_Co_0.2_Fe_0.8_O_2.5_ + 0.19O_2_ (3)

The relative mass change Δm can be calculated using the following equation:

(4) $$\Delta m=\frac{M_{2}-M_{1}}{M_{1}}$$

where *M*_1_ is the molar mass of La_0.6_Ca_0.4_Co_0.2_Fe_0.8_O_2.88_ and *M*_2_ is the molar mass of La_0.6_Ca_0.4_Co_0.2_Fe_0.8_O_2.5._

The maximum theoretical mass change (Δm) for La_0.6_Ca_0.4_Co_0.2_Fe_0.8_O_2.88_, calculated from equation 4, is −3.01%. From the TGA (Figure 5a), the observed mass change for LCCF_6428 up to 1100 °C is −1.32%. After a prolonged exposure of 25 h, the mass change increases to –2.66%. A slight weight gain can be noticed during the isothermal step around the 500–700 min range. This weight gain is unexpected, and the exact origin is currently unknown to us, but it could be due to minor irregularities experienced by the device during the measurement. Despite the mass change being lower than the estimated theoretical value, traces of secondary phases (Ruddlesden–Popper-type oxide) potentially alongside binary oxides can still be seen in the XRD pattern (Figure 5b). The emergence of secondary phases coupled with the partial phase transitions above 800 °C [38] can be indicative of a structural degradation.

**Figure 6:**
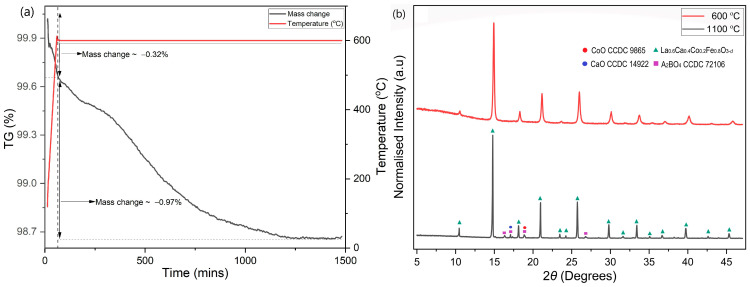
(**a**) Mass change using TGA noticed in LCCF_6428 powder sample up to 600 °C in 95 vol.% Ar-5 vol.% H_2_. (**b**) XRD plots of LCCF-6428 powder sample after exposure to 95 vol.% Ar-5 vol.% H_2_ at 600 °C and 1100 °C (Mo-*Kα*_1_).

**Figure 7:**
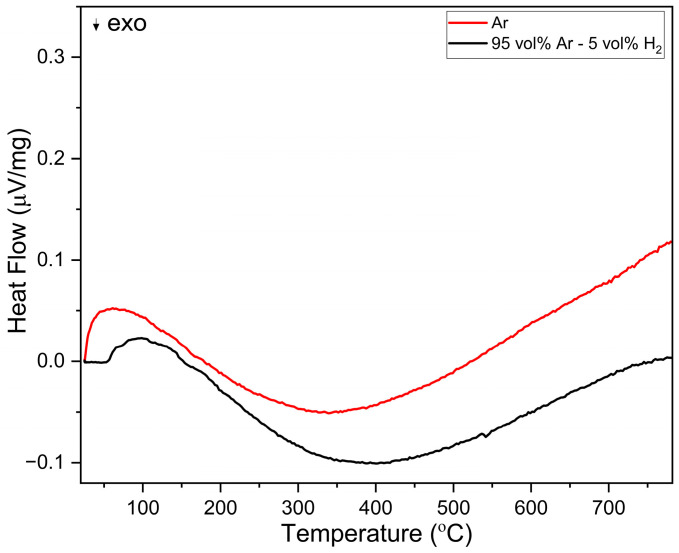
DSC analysis of LCCF-6428 in Ar and 95 vol% Ar-5 vol% H_2_ up to 800 °C.

The behaviour up to 600 °C was further investigated isothermally over a duration of 25 h (Figure 6a). During heating, LCCF_6428 showed a significantly lower mass change of −0.32%. After the 25 h exposure to 95 vol. % Ar-5 vol. % H_2_, in the first 20 h, the mass change increased to −1.29%, but afterwards, it stayed constant. From the TGA, the observed mass change for LCCF_6428 (−1.29% at 600 °C for 25 h) is notably lower than the predicted tolerable value of −3.01% (Figure 6a). The XRD data (Figure 6b) are in tandem with mass change observations and do not indicate any structural degradation. The DSC measurements (Figure 7) in Ar and 95 vol.% Ar-5 vol.% H_2_ do not reveal solid evidence for the presence of a first-order phase transition up to 800 °C.

It must, however, be noted that here the membrane was exposed to extremely harsh conditions in an isolated environment of H_2_. The actual process is to be carried out in the CO_2_/H_2_ plasma system where the presence of oxygen-containing species (such as H_2_O, O_2_, and O*) is expected. It was assumed that the oxygen-containing species can compensate for the oxygen lost due to exposure to H_2_ and, in turn, heal the membrane. This can play a major role towards enhancing the stability of the membrane beyond 600 °C in a H_2_-containing plasma. To test this assumption, a preliminary plasma exposure test was carried out, where the membrane was exposed to a CO_2_/H_2_ plasma (Section 3.5).

### 3.5. Preliminary Plasma Test

**Figure 8:**
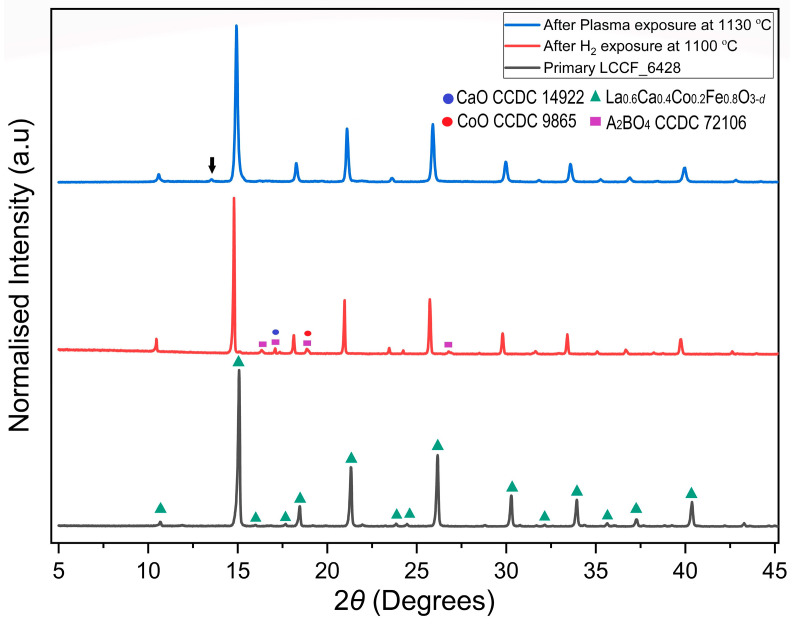
Comparison of XRD patterns of sintered primary LCCF_6428 membrane vs. H_2_ exposure at 1100 °C vs. plasma-exposed LCCF_6428 membrane at 1130 °C (Mo-*Kα*_1_). The downward arrow in the plasma-exposed LCCF_6428 membrane at 2*θ* ≈ 13° represents the minor impurity peak.

The LCCF membrane was exposed to a CO_2_/H_2_ plasma at 1130 °C for 1 h. The XRD pattern (Figure 8) after the CO_2_/H_2_ plasma exposure at 1130 °C appears to match well with the primary sample without any presence of major impurities, and it does not indicate structural degradation, unlike isolated H_2_ exposure at 1100 °C, which was noticed earlier in the thermal analysis (Section 3.4). The minor impurity peaks present in the primary sample at lower angles also cannot be found in the plasma-exposed sample, except the one present at 2*θ* ≈ 13°.

**Figure 9:**
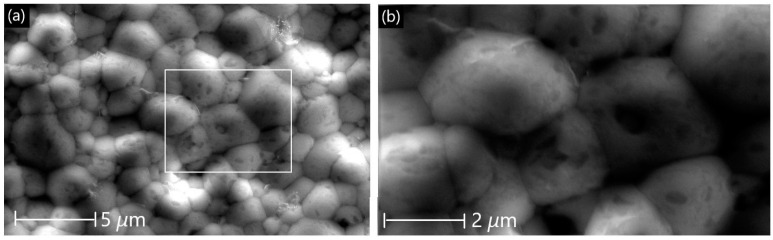
SEM surface micrographs of LCCF_6428 after exposure to plasma at 1130 °C. (**a**) Lower magnification (scale bar: 5 µm) and (**b**) higher magnification (scale bar: 2 µm).

Figure 9 shows the morphology of the membrane after the plasma test. The grains show a compact arrangement resembling that of the primary membrane (Figure 3). The presence of pores is very minimal and isolated, and they do not appear to coalesce with each other.

**Figure 10:**
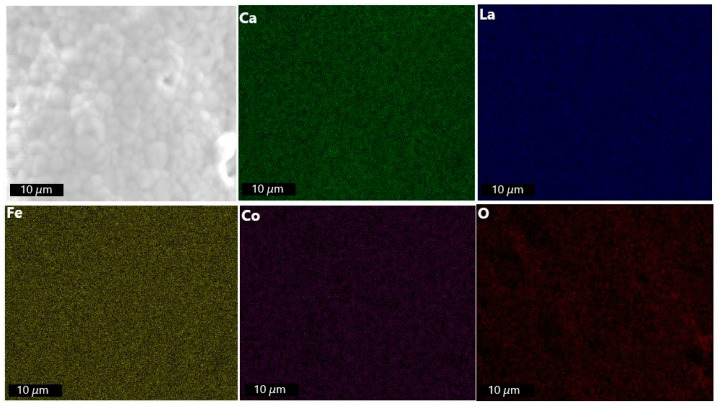
EDXS mapping of LCCF_6428 membrane (non-polished) after exposure to plasma at 1130 °C.

The EDXS mapping (Figure 10) of the membrane after plasma exposure points towards the homogeneous distribution of the elements being maintained compared to the primary membrane (Figure 4). This is supported by the EDXS elemental analysis, which can be accessed in the supplementary section (Table S2).

The preliminary results are a positive indication towards our assumption of oxygen-containing species present in the CO_2_/H_2_ plasma playing a major role in healing the membrane against H_2_ exposure. And, this could indicate an increased atmospheric tolerance of at least up to 1130 °C instead of 600 °C in the given conditions.

### 3.6. Oxygen Permeation Performance

The performance of the membrane can be assessed by its oxygen permeation capability. This test allows us to measure the oxygen diffusion of the membrane through its percolation network formed by the oxygen vacancies. Previously, Chen et al. [19] synthesised La_0.6_Ca_0.4_Co_0.5_Fe_0.5_O_3–*d*_ (thickness ~1 mm) using the co-precipitation method and recorded a permeability of 0.5 mL∙min^−1^∙cm^−2^∙mm with a relative leakage of 30–35%. It is pertinent to mention that this membrane has a Co:Fe ratio of 1:1. However, in this work, the LCCF membrane La_0.6_Ca_0.4_Co_0.2_Fe_0.8_O_3–*d*_ (thickness ~0.94 mm) prepared using USS has a 1:4 Co:Fe ratio, thereby using a 30% lower amount of cobalt. An oxygen permeability of 0.41 mL∙min^−1^∙cm^−2^∙mm was recorded for the membrane (Table 3) with a relative leakage of only 9–12%, which helps with improving the gas tightness of the membrane. The permeability is 80% of the one prepared with co-precipitation but with a significantly lower leakage and lower cobalt content used.

The leakage during the measurement can be associated to (i) how firmly the membrane is attached to the alumina tubes using a ceramic paste and (ii) the amount of N_2_ in the permeate gas [33]. Oxygen can also traverse through pores if the membrane is not dense enough. However, given the relative density of our membrane is ~89%, the influence of a small number of pores is less significant. The effect of the leakage flow, in cases of very high leakage, on the oxygen partial pressure gradient can also affect the oxygen permeation. This requires the determination of the individual oxygen partial pressures on the feed and sweep side. However, the current construction of the permeation setup does not allow for measuring these oxygen partial pressures. This is planned for future studies.

## 4. Discussion

The selection of synthesis procedure is quite significant for this work. Conventional routes, such as sol–gel and co-precipitation, are good enough to produce membranes with similar performance, as can be noticed in previous works [6,19], but they do not meet the continuity and resource efficiency goals that are demanded in the future. Meanwhile, ultrasonic spray synthesis helps tackle these challenges. It provides a fine crystalline powder product that is easier to process for hollow fibre membrane production [6]. And, its scope for upscaling is boosted by the fact that it implements a continuous route to the product, hence providing a reliable production route instead of a batch system.

The main requirements for the membrane in plasma-assisted CO_2_ conversion are good thermal stability against CO_2_ and H_2_ atmospheres along with a decent oxygen permeability [23,24,25,26]. LCCF_6428 has been shown to be capable of fulfilling these requirements in this study. It performed well in the tested plasma conditions, providing sufficient oxygen permeability for it to be considered for industrial use. The selection of Co at the *B* site ahead of other possible substitutes mostly from late transition metals, such as Cu, Ni, Zn, and Mn, is very interesting. Although using an oxygen transport membrane containing Co can be a challenge economically due to its criticality, it usually performs better in terms of oxygen permeation when compared with the other substitutes owing to the membrane’s decent oxygen diffusion through the bulk and better surface exchange kinetics, comparatively [39]. The challenges posed by cobalt’s criticality and lanthanum’s rare earth nature were dealt with through our substitution approach that enabled the use of the critical elements in minimum possible amounts.

The thermal analysis of LCCF in reducing conditions of H_2_ presented a worst-case scenario detailing the structural degradation that could happen to the membrane if exposed to very harsh conditions (Figure 5a,b) and, consequently, a decline in the performance is expected. From the TGA data, it can also be deduced that lowering the cobalt content in LCCF is favourable for improved H_2_ tolerance, as observed in case of LCCF_6428, without sacrificing the performance of the membrane in terms of oxygen transport. The usage of CO_2_/H_2_ plasma can prove beneficial with regards to enhancing the required atmospheric tolerance beyond 600 °C, going up to 1130 °C at least. It provides very unique conditions that can help the membrane structure to stay intact by healing the membrane with the oxygen-containing species, such as H_2_O, O_2_, and O* present in the plasma, as can be understood from the preliminary plasma test results.

The oxygen permeation results showed the performance of the membrane synthesised with USS to be on par with the conventionally produced methods. The oxygen permeation in mixed ionic electronic conducting membranes has two rate-determining steps: (i) bulk diffusion of electrons and ions and (ii) the surface exchange of oxygen [23,24]. The dominant behaviour of bulk diffusion in LCCF membranes can be tackled by reducing the thickness of the membranes, such as by developing hollow fibres (thickness 0.2 mm) instead of pellets (thickness 1 mm) [6].

## 5. Conclusions

La_0.6_Ca_0.4_Co_0.2_Fe_0.8_O_3–*d*_ membranes were synthesised successfully with the help of ultrasonic spray synthesis. This technique is an effective way of producing the La_0.6_Ca_0.4_Co_0.2_Fe_0.8_O_3–*d*_ membrane materials at a much faster rate than the conventional techniques. It also has a promising scope to be a continuous process, which can prove very beneficial while upscaling. In addition to CO_2_ tolerance, the H_2_ tolerance of the La_0.6_Ca_0.4_Co_0.2_Fe_0.8_O_3–*d*_ membrane was established at 600 °C for 25 h. In the plasma system, the required atmospheric tolerance has the potential to last up to at least 1130 °C, as per the preliminary results. The oxygen permeation results of the membranes were satisfactory. The membrane exhibited an oxygen permeability of 0.41 mL∙min^−1^∙cm^−2^∙mm, with reduced usage of cobalt.

## Figures and Tables

**Table 1:** ICP-OES analysis of LCCF_6428 (±indicates the error bar).

Element	Theoretical wt% (Approx.)	Primary Synthesis—wt% (Average)
La	40.89	40.80 ± 0.05
Ca	7.86	7.63 ± 0.06
Co	5.78	5.67 ± 0.04
Fe	21.92	22.40 ± 0.10

**Table 2:** HGE analysis of LCCF_6428 (±indicates the error bar).

Element	Theoretical wt% (Approx.)	Primary Synthesis wt% (Average)
O	23.55	22.67 ± 0.26

**Table 3:** Oxygen permeation data of LCCF membranes.

Membrane	Oxygen Permeation Flux (mL·min^−1^·cm^−2^)	*d* (mm)	Permeability (mL·min^−1^·cm^−2^·mm)	Relative Leakage
LCCF-6428 (USS)	0.426	0.94	0.40	9–12%
LCCF-6455 (CP [19])	0.500	1.00	0.50	30–35%

## Data Availability

The data are available upon request to the corresponding authors. The diffraction data collected at ESRF can be accessed as given in reference [35].

## Supplementary Materials

The following supporting information can be downloaded at: https://www.mdpi.com/article/10.3390/membranes13110875/s1, Figure S1: The experimental setup of USS, Figure S2: The schematics (left) and the nebulising unit image (right), Figure S3: Geometry of the quartz glass tube in the furnace, Figure S4: The extraction system of USS, Figure S5: XRD patterns of LCCF_6428 (primary, plasma exposed) vs calculated reference pattern of LaFeO_3_ (CCDC-28255), Figure S6: XRD patterns of LCCF_6428: primary vs permeation test vs plasma test. Figure S7: SEM micrograph of LCCF_6455 after exposure to 95 vol.% Ar-5 vol.% H_2_ at 1100 °C, Table S1: Summarised Rietveld refinement parameters, Table S2: EDXS analysis of LCCF_6428, Table S3: EDXS analysis of LCCF_6455, Table S4: Gas qualities used for TGA, plasma exposure, and oxygen permeation measurements.
