# Supplementary material for: Hydrogen-Tolerant La0.6Ca0.4Co0.2Fe0.8O3–d Oxygen Transport Membranes from Ultrasonic Spray Synthesis for Plasma-Assisted CO2 Conversion
Source: Membranes (Basel). 2023 Nov 7;13(11):875. doi: 10.3390/membranes13110875 (PMC10673528; doi:10.3390/membranes13110875)
Supplement: Supplementary file 1 [file membranes-13-00875-s001.zip › membranes-2609400-supplementary.pdf]

Electronic Supplementary Information

Hydrogen Tolerant $La_{0.6}Ca_{0.4}Co_{0.2}Fe_{0.8}O_{3-d}$ Oxygen Transport Membranes from Ultrasonic Spray Synthesis for Plasma-assisted CO_2-Conversion

Aasir Rashid [1*], Hyunjung Lim [1], Daniel Plaz [2], Giamper Escobar Cano [3], Marc Bresser [4], Katharina-Sophia Wiegers [4], Giorgia Confalonieri [5], Sungho Baek [1], Guoxing Chen [6], Armin Feldhoff [3], Andreas Schulz [4], Anke Weidenkaff [1,6] and Marc Widenmeyer [1,*]

1 Research Division of Materials & Resources, Technical University of Darmstadt, Peter-Grünberg-Str. 2, 64287 Darmstadt, Germany; hyunjung.lim@stud.tu-darmstadt.de (H.L.); sungho.baek@stud.tu-darmstadt.de (S.B.); anke.weidenkaff@mr.tu-darmstadt.de (A.W.)

2 Institute for Materials Science, University of Stuttgart, Heisenbergstr. 3, 70569 Stuttgart, Germany; daniel.plaz@t-online.de

3 Institute of Physical Chemistry and Electrochemistry, Leibniz University Hannover, Callinstr. 3a, 30167 Hannover, Germany; giamper.escobar@pci.uni-hannover.de (G.E.C.); armin.feldhoff@pci.uni-hannover.de (A.F.)

4 Institute of Interfacial Process Engineering and Plasma Technology (IGVP), University of Stuttgart, Pfaffenwaldring 31, 70569 Stuttgart, Germany; marc.bresser@igvp.uni-stuttgart.de (M.B.); katharina.wiegers@igvp.uni-stuttgart.de (K.-S.W.); andreas.schulz@igvp.uni-stuttgart.de (A.S.)

5 ESRF—European Synchrotron Research Facility, 71 Avenue des Martyrs, 38043 Grenoble, France; giorgia.confalonieri@esrf.fr

6 Fraunhofer Research Institution for Material Recycling and Resource Strategies IWKS, Brentanostr. 2A, 63755 Alzenau, Germany; guoxing.chen@iwks.fraunhofer.de

* Correspondence: aasir.rashid@mr.tu-darmstadt.de (A.R.); marc.widenmeyer@mr.tu-darmstadt.de (M.W.)

S1. Experimental Setup of Ultrasonic Spray Synthesis (USS)

The USS experimental setup can be separated into three main parts, the nebulising unit, tube furnace, and the extraction system. The complementary instruments, a gas flow meter, and a two-channel resistance thermometer are connected to the respective units to control the experimental system. The whole experimental setup is shown in Figure S1.

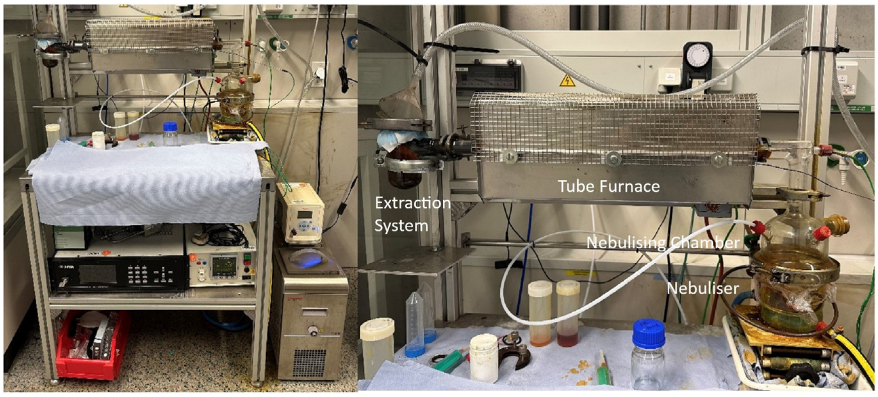

Figure S1: The experimental setup of USS.

1.1. Nebulising Unit

The nebulising unit consists of two separated parts namely the transducer chamber and the nebulising chamber. The schematics are shown in the Figure S2.

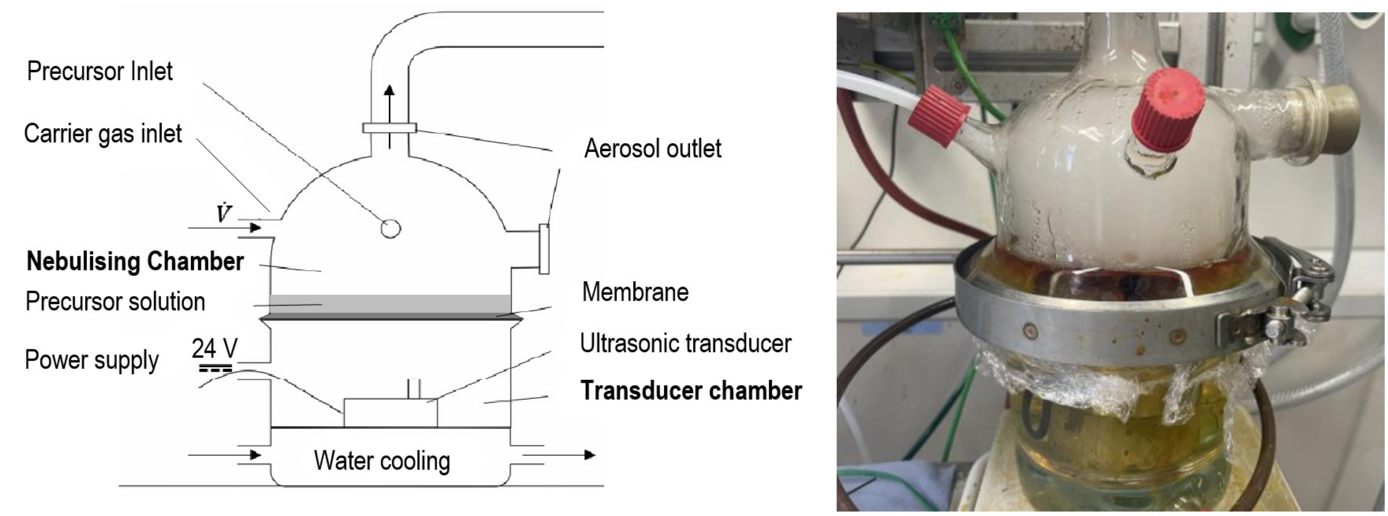

Figure S2: The schematics (left) and the nebulising unit image (right).

As shown in Figure S2, the nebulising unit can contain the precursor solution and enables nebulisation of solution by an ultrasonic transducer located in the transducer chamber situated below the nebulising chamber. The two chambers are separated by a polymeric membrane to prevent corrosion and failure of ultrasonic transducer.

The ultrasonic transducer, a Fogstar 100 from Seliger GmbH, Villingen-Schwenningen, Germany, is a commercial product which can generate ultrasonic waves with 24 V DC power supply at a power output of 28.8 W. Generated ultrasonic waves have a frequency of 1.7 MHz. The transducer chamber is filled with water, which can be cooled from below by a cooling water flow. The generated ultrasonic waves pass through the water inside the transducer chamber and reach the membrane between the two chambers, the precursor solution can be nebulised by conveyed ultrasonic waves [1]. When nebulisation of the precursor solution occurs, the generated precursor aerosols are transported into the aerosol outlet and go through the tube furnace with the carrier gas flow.

1.2. The tube furnace

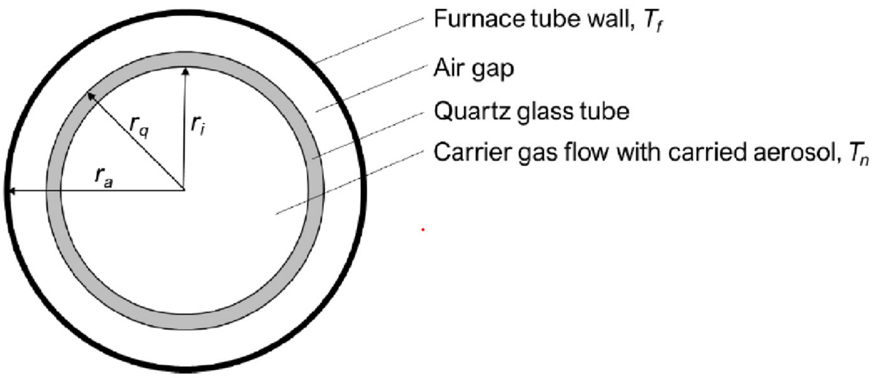

Figure S3: Geometry of the quartz glass tube in the tube furnace. (Diameter of the quartz glass tube inside the furnace, r_q = 36.21 mm)

The tube furnace is a Type LOBA 1200-45-400 made by HTM Reetz GmbH, Berlin, Germany. It has a maximum upper temperature limit of 1200 °C, and tube length is 400 mm with a diameter (r_a) of 45 mm. The temperature of the tube furnace is controlled by the thermocouple connected with the furnace, and the maximum furnace temperature is measured by an additional thermocouple due to the deviation between the temperature inside of the quartz glass tube and the furnace itself. The scheme of cross section of the quartz glass tube is described in Figure S3.

1.3. The extraction system

The extraction unit consists of a 250 ml glass vessel with a glass joint which can be connected to the quartz glass tube in the furnace. The diameter of the top of the vessel is 5 cm, and it is covered by a commercial surgical mask as a filter, held by an aluminium framed O-ring and closed by a metal clamp. The filter mask has three layers where the products are collected, allowing the carrier gas to pass through the filter to the exhaust and providing low back pressure in the whole system. The experimental setup of the extraction unit is shown in Figure S4.

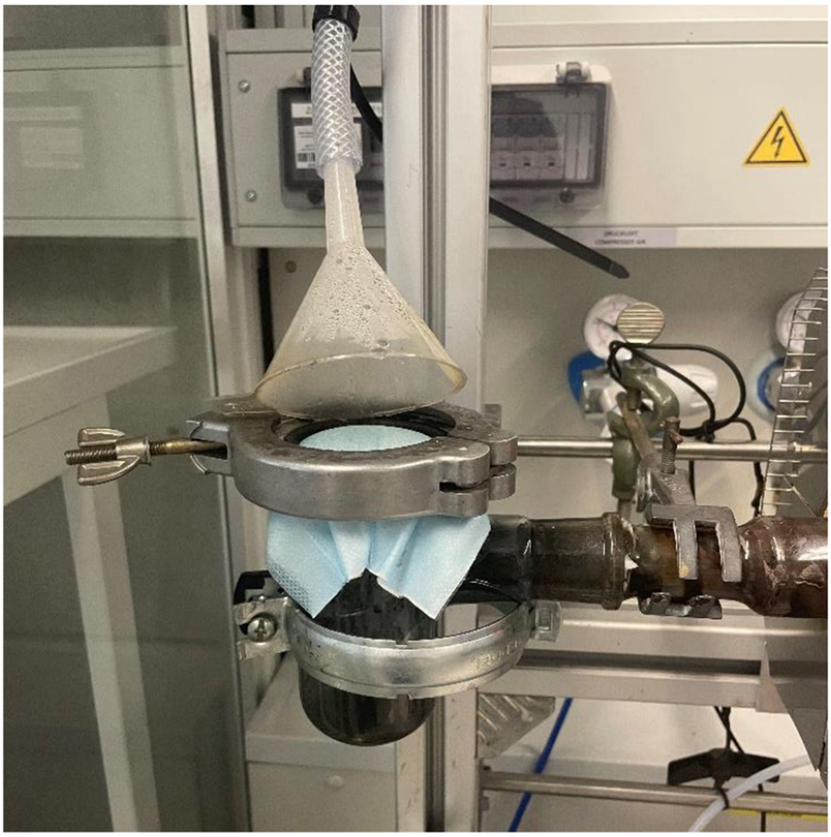

Figure S4: The extraction system of USS.

1.4. Complementary Equipment

The complementary instruments are required to control the experimental system of USS. A two-channel resistance thermometer, a Eurotherm 3504 made by Eurotherm Deutschland GmbH, Limburg, Germany, is connected to the tube furnace to control the temperature, and the temperature in the tube furnace is observed with a Type K thermocouple. The temperature between the outlet of the furnace and the inside of the tube has variance, therefore, the additional thermometer is used to measure the maximum temperature of the tube furnace inside, where the reaction occurs. The additional thermometer is a YC- 727U data logger thermometer manufactured by Maxtech, which is a two-channel thermometer combined with two Type K thermocouples. The carrier gas flow is controlled by the gas flow meter, a mass flow controller MKS instruments Type 1179 made by MKS Instruments Germany GmbH, München, Germany. The maximum gas flow that can be provided to the system is 6000 sccm.

S2. XRD

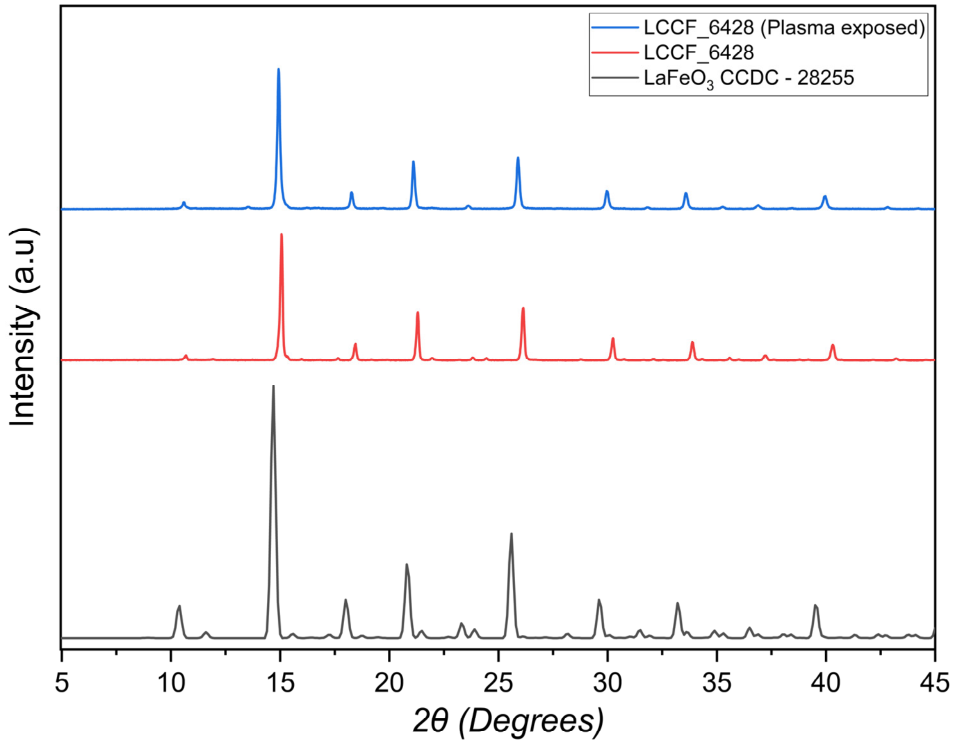

Figure S5: XRD patterns (λ = 0.7093 Å) of LCCF_6428 (primary, plasma exposed) vs calculated reference pattern of $LaFeO_3$ (CCDC-28255).

The XRD patterns of LCCF_6428 across the mentioned stages match with the reference $LaFeO_3$ with the crystal structure resembling with that of a perovskite-type oxide. There are no indications of a secondary phase being present.

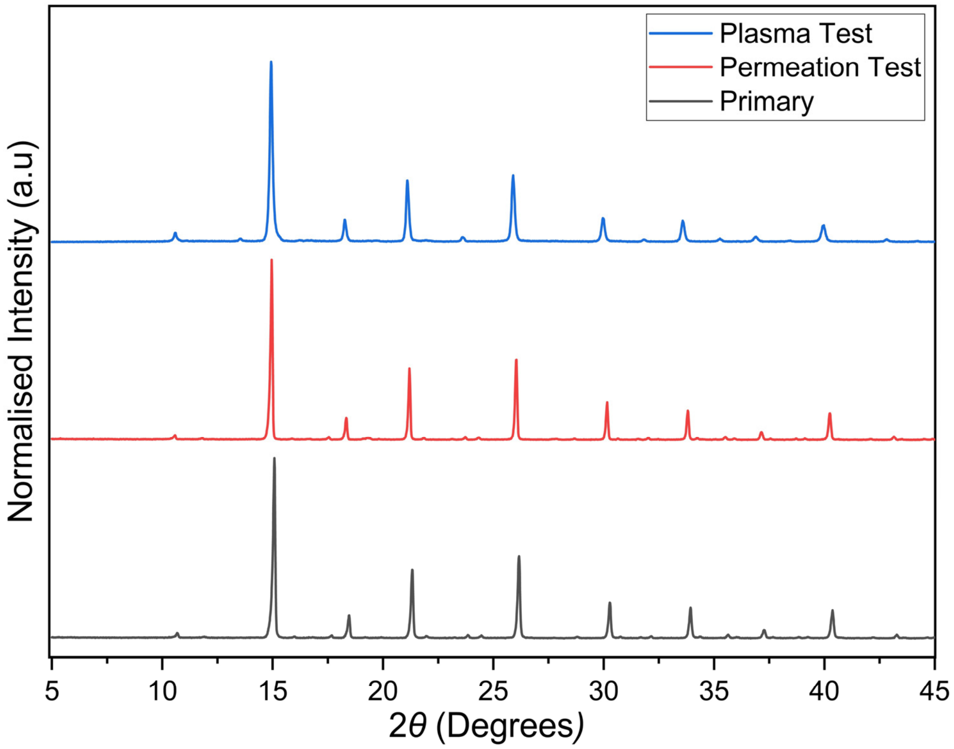

Figure S6: XRD patterns of LCCF_6428: primary vs permeation test vs plasma test (λ = 0.7093 Å).

Figure S6 gives a comparison between the recorded XRD patterns of LCCF_6428 after undergoing permeation test and plasma test. The XRD patterns across all the stages seem to match well with the primary LCCF_6428 without any presence of a secondary phase.

Rietveld Refinements

Table S1. Summarised Rietveld refinement parameters.

**Parameter**	**LCCF_6428**
**Space Group**	*Pnma*
**Unit cell parameters (*a,b,c*) (Å)**	5.4688(1)<br>7.7419(2)<br>5.4895(1)
**Unit cell Volume (Å)^3**	232.419(7)
**$B_{iso,overall}$ (Å^2)**	0.66(1)
**x(La, Ca)**	0.4816(1)
**z(La, Ca)**	0.0037(2)
**x(O(1))**	0.2196(8)
**y(O(1))**	0.0342(6)
**z(O(1))**	0.272(1)
**x(O(2))**	0.5022(7)
**z(O(2))**	0.563(1)
**w(La)**	0.71(2)
**w(Ca)**	0.29(2)
**R_p (%)**	5.62
**R_{wp} (%)**	9.01
**R_{exp} (%)**	3.62
χ^2	6.420
**Bragg *R*-factor (%)**	3.88
**Density (g/cm^3)**	5.826

S3. SEM-EDXS

In contrast to the Rietveld refinements, the EDXS analysis (Table S2) indicated a chemical composition rather close to the theoretically expected. The small variation around the respective average measurement values points to uniformity in the distribution of the elements within the given special resolution. The obtainable EDXS analysis results are in sufficient agreement with the ICP-OES. The oxygen content was excluded from the EDXS analysis, since due to the low electron count of oxygen, a reliable quantification is very challenging. Instead, the theoretical values were calculated based on a hypothetical "$La_{0.6}Ca_{0.4}Co_{0.2}Fe_{0.8}$" compound.

TableS2. EDXS analysis of LCCF_6428 after synthesis and plasma exposure (cations only; ± indicates the error bar).

Element	Theoretical wt% (approx.)	Primary Synthesis wt% (Average)	Plasma Exposure wt% (Average)
La	53.48	54.64 ± 0.01	54.12 ± 0.51
Ca	10.29	11.01 ± 0.04	9.69 ± 0.09
Co	7.56	7.25 ± 0.03	7.28 ± 0.06
Fe	28.67	27.11 ± 0.05	28.91 ± 0.24

To study any potential compositional change in the membrane disc after plasma exposure, EDXS analysis (Table S2) was used. No obvious composition change was observed after the plasma exposure.

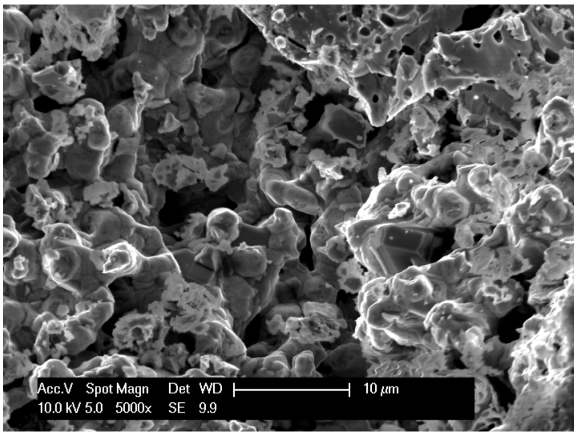

Fig. S7: SEM micrograph of LCCF_6455 after exposure to 95 vol.% Ar-5 vol.% H_2 at 1100 °C.

The morphology of LCCF_6455 after exposure to 95 vol.% Ar-5 vol.% H_2 at 1100 °C revealed a disjoint microstructure with certain grains subjected to either recrystallisation or indicative of melting.

TableS3. EDXS analysis of LCCF_6455 after synthesis and H_2 exposure at 1100 °C (cations only; ± indicates the error bar).

Element	Theoretical wt% (approx.)	Primary Synthesis wt% (Average)	H_2 Exposure wt% (Average)
La	53.16	55.85 ± 0.40	56.17 ± 0.54
Ca	10.23	10.10 ± 0.07	8.79 ± 0.09
Co	18.80	19.89 ± 0.13	7.76 ± 0.06
Fe	17.81	18.85 ± 0.05	27.28 ± 0.22

The EDXS analysis shows LCCF_6455 being highly Co deficient after H_2 exposure at 1100 °C.

S4. Gases used for measurements

Table S4. Gas qualities used for TGA, plasma exposure, and oxygen permeation measurements.

Gas	Composition	Additional components / ppm	Purity / %
**For Oxygen Permeation**			
Synthetic Air	O_2: 20 %<br>N_2: 80 %	$H_2O \leq 2$<br>$CO_2 \leq 1$<br>$CO \leq 1$<br>Hydrocarbons ≤ 0.1<br>$NO_x \leq 0.1$	-
Neon	Ne	$N_2 \leq 5$<br>$O_2 \leq 2$<br>$H_2O \leq 3$<br>Hydrocarbons ≤ 0.2<br>He < 20	≥ 99.995
Carbon Dioxide	CO_2	$N_2 \leq 25$<br>$O_2 \leq 15$<br>$H_2O \leq 5$<br>Hydrocarbons ≤ 1<br>CO < 1	≥ 99.995
**For Thermal Analysis**			
Argon -Hydrogen Mixture	Ar: 95 vol%<br>H_2: 5 vol%	$H_2O \leq 5$<br>$O_2 \leq 5$<br>Hydrocarbons ≤ 0.5	≥ 99.999
Argon	Ar: 100%	-	≥ 99.999%
**For Plasma**			
Hydrogen	H_2: 100%	-	≥ 99.999%
Carbon Dioxide	CO_2	$N_2 \leq 20$<br>$O_2 \leq 10$<br>$H_2O \leq 5$<br>Hydrocarbons and CO ≤ 10	≥ 99.995%

Reference

1. Fu, J., Daanen, N. N., Rugen, E. E., Chen, D. P., & Skrabalak, S. E. (2017). Simple reactor for ultrasonic spray synthesis of nanostructured materials. *Chemistry of Materials*, *29*(1), 62-68. https://doi.org/10.1021/acs.chemmater.6b02660.
